# Therapeutic manipulation of gut microbiota by polysaccharides of *Wolfiporia cocos* reveals the contribution of the gut fungi-induced PGE_2_ to alcoholic hepatic steatosis

**DOI:** 10.1080/19490976.2020.1830693

**Published:** 2020-10-27

**Authors:** Shanshan Sun, Kai Wang, Li Sun, Baosong Cheng, Shanshan Qiao, Huanqin Dai, Wenyu Shi, Juncai Ma, Hongwei Liu

**Affiliations:** a State Key Laboratory of Mycology, Institute of Microbiology, Chinese Academy of Sciences, Beijing, China; b School of Life Sciences, University of Science and Technology of China, Hefei, P. R. China; c Savaid Medical School, University of Chinese Academy of Sciences, Beijing, P. R. China; d Microbial Resource and Big Data Center, Institute of Microbiology, Chinese Academy of Sciences, Beijing, China

**Keywords:** *Wolfiporia cocos* polysaccharides, alcoholic liver diseases, gut mycobiota, *Meyerozyma guilliermondii*, fungi-induced PGE_2_

## Abstract

Alcohol abuse and alcoholic liver diseases (ALD) have been worldwide spread. Chronic alcoholism-induced overgrowth of intestinal bacteria and fungi together with the enteric dysbiosis are important pathogenic mechanisms in ALD. We demonstrated that the water-insoluble polysaccharides (WIP) from *Wolfporia cocos* effectively ameliorated the hepatic inflammatory injury and fat accumulation through modulating gut microbiota in mice with alcoholic hepatic steatosis (AHS). Oral administration of WIP significantly enhanced the ratio of Firmictues to Proteobacteria, increased the abundance of Lachnospiraceae including Ruminoclostridum and unidentified_clostridials, and inhibited the ethanol-induced fungal overgrowth. Treatment with WIP activated the PPAR-γ signaling and reduced the inflammation in the colonic epithelia cell, facilitating a hypoxic state that suppresses the overgrowth of fungi and Proteobacteria in the gut. In addition, we found an overwhelming increase of the commensal fungus *Meyerozyma guilliermondii* in the feces of mice with AHS by culturing and ITS sequencing. Inoculation of *M. guilliermondii* into fungi-free mice aggravated the features of AHS. *M. guilliermondii* was found to generate PGE_2_ by biotransformation of arachidonic acid. Furthermore, the gut fungi (*M. guilliermondii*)-induced PGE_2_ production in the liver was confirmed as one of the mechanisms in the chronic AHS. The current study supports the manipulation of the gut microbiota (bacteria and fungi) as an effective and alternative strategy for alleviating ALD.

## Introduction

Alcoholic liver diseases (ALD) have become one of the most prevailing chronic liver diseases worldwide. More importantly, alcohol-attributable death accounts for 5.3% of worldwide death in 2016 according to the global status report on alcohol and health released by WHO in 2018. The pathogenesis of alcoholic liver diseases is multifactorial and complicated. Recently, increasing evidence has revealed a non-negligible and causative association between gut dysbiosis and the development of ALD. Chronic consumption of alcohol causes damages on intestinal tract integrity and alteration in gut microbiota.^1–4^ Due to high susceptibility to ethanol, the intestinal bacteria community is greatly influence by alcohol consumption. It was found that the abundance of Proteobacteria was increased while the abundance of Firmicutes and Bacteroides were decreased in the gut microbiome of ALD animals and patients with ALD without cirrhosis.^5–7^ Correspondingly, the significant elevation of endotoxins, the change of bile acids and the decrease of short-chain fatty acids were observed in the gut of ALD animals or patients with ALD.^8,9^ In comparison with the well-defined contribution of the intestinal bacteria to ALD, the roles and mechanisms of commensal fungi in the development of ALD are initially investigated. The alcohol-enhanced fungal community in gut along with fungi-induced systemic immune response and the fungal β-glucan-activated inflammation response have been demonstrated to be associated with the development of ALD.^10,11^

The interactions between or among the intestinal bacteria, fungi, and virus are very important for maintaining the homeostasis of gut microbiota. Several studies demonstrated that the obligate anaerobic bacteria in the phylum of Firmicutes played important roles in controlling the expansion of potentially pathogenic bacteria in the phylum of Proteobacteria and the colonization of commensal fungi in the gut.^12,13^ These findings as well as the evidence that the alcohol-induced bacterial dysbiosis and fungal overgrowth contributed to the liver inflammation and fat deposition suggest that the alcohol-caused decrease of Firmucutes may be one of the principle factors involving in the pathogenesis of ALD. Therefore, approaches that specifically promote the growth of bacteria in the phylum of Firmicutes are expecting for the treatment of ALD. In an early work, the prebiotic fructooligosaccharides that stimulate the growth of *Lactobacilli* and *Bifdobacteria* were confirmed to ameliorate the alcohol-induced liver damage in mice.^5^ As the edible and medicinal mushroom, the sclerotium of *W. cocos* is widely used in traditional Chinese medicine due to its diuretic, sedative, and tonic effects. In our early research, oral administration with the water-insoluble polysaccharide (WIP) from *Wolfiporia cocos* (previously named as *Poria cocos*) increased the abundance of butyrate-producing bacteria in the phylum of Firmicutes in *ob/ob* mice,^14^ which indicates its potentially beneficial effects on ALD. WIP is a (1-3)-β-D-glucan with an average molecular weight of 4.486 × 10^6^ Da as identified by NMR and SEC-RI-MALLS analyses. So far, the effect of *W. cocos* on ALD is not investigated.

In the current work, by using mice with alcoholic hepatic steatosis as a model, we demonstrated the therapeutic efficacies of WIP on ethanol-induced liver fat accumulation and inflammation, confirmed the beneficial effects of WIP on the ethanol-induced gut dysbiosis, and revealed an association of the commensal fungus *Meyerozyma guilliermondii* with ALD as well as the contribution of fungi-induced PGE_2_ to the development of ALD.

## Results

### Oral treatment with WIP alleviates chronic ethanol feeding-induced hepatic injury and steatosis

**Figure 1.**
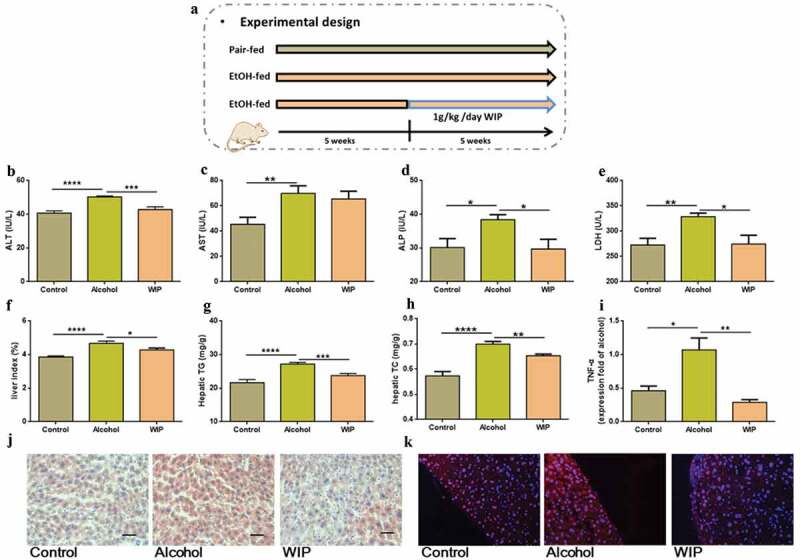
Oral treatment with WIP alleviates chronic ethanol feeding-induced hepatic injury and steatosis

After five weeks of ethanol feeding, C57BL/6 J mice were separated into two groups and then treated with the vehicle and the polysaccharides (WIP) at the dose of 1.0 g/kg/day (the equivalent dose of 0.11 g/kg/day in humans) from *W. cocos* for another five weeks while continuing ethanol feeding (Figure 1a). The control group was given with isocaloric control diet. The water-insoluble polysaccharides were prepared as described in our early work.^14^ In comparison with the alcohol group, oral treatment with WIP significantly alleviated liver injury, as indicated by ALT, ALP and LDH(Figure 1b-e). In addition, WIP supplementation dramatically reduced the alcoholic hepatic steatosis, as confirmed by decreases in the liver index and the levels of the hepatic triglyceride (TG) and the hepatic total cholesterol (TC) (figure 1f-h). The oil-red staining further indicated the reduction of liver fatty accumulation (Figure 1j). In an early work, monocyte chemotactic protein 1 (MCP-1) was reported to promote macrophage activation, proinflammatory response, and hepatic steatosis.^15^ Herein, we found that WIP treatment effectively reduced the MCP-1 expression in the liver of mice receiving chronic ethanol consumption by an immune histological detection (Figure 1k).

(a) Experimental design. (b) The level of plasma alanine aminotransferase (ALT). (c) The level of plasma aspartate aminotransferase (AST). (d) The plasma levels of alkaline phosphatase (ALP). (e) The level of plasma lactate dehydrogenase (LDH). (f) Liver index. (g) The level of hepatic triglyceride (TG). (h) The level of hepatic total cholesterol (TC). (i) The expression of TNF-α in liver. (j) Representative picture of liver sections stained with oil-red. (k) Representative picture of liver sections with MCP-1 immunofluorescence staining. (b-h) N = 10 per group, (i) N = 5 per group, (j-k) N = 3 per group. Control: mice received isocaloric liquid diet instead of ethanol. Alcohol: mice fed with ethanol diet. WIP: mice fed with an ethanol diet supplemented with a water-insoluble polysaccharide from *W. cocos*. Data are presented as the mean ± standard error of the mean (SEM). Statistical analysis was done using one-way ANOVA followed by the Tukey post hoc test. Compare to Alcohol: * *P* < .05; ** *P* < .01; *** *P* < .001; **** *P* < .0001.

### Amelioration of the ethanol-induced gut dysbiosis by WIP treatment

**Figure 2.**
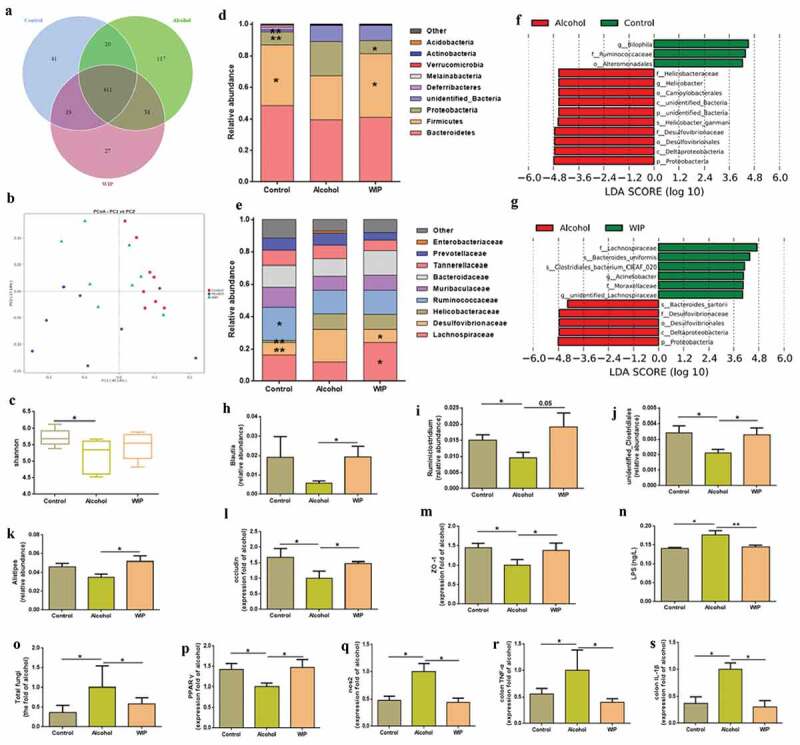
WIP treatment ameliorates the ethanol-induced gut dysbiosis

To investigate the impact of WIP on gut microbiota of the mice with ethanol feeding-induced hepatic injury and steatosis, we analyzed the composition and abundance of gut microbiota by high-throughput sequencing of the 16S rRNA gene V3 and V4 regions of the cecal contents of these mice. 673 OTUs were obtained at a similarity threshold of 97%. Average of OTUs for each group and overlap were performed using Veen diagram (Figure 2a). As to the community similarity, weighted uniFrac-based principal coordinates analysis (PCoA) revealed a distinct clustering of microbiota composition for each group (Figure 2b). Meanwhile, the chronic ethanol administration significantly decreased the alpha-diversity of gut microbiota as indicated by Shannon index (Figure 2c). In contrast, treatment with WIP led to a non-significant increase of the Shannon index, as compared with that of alcohol group. The changes in the gut bacterial community of ethanol-fed mice observed in this work were similar to those of previous investigations.^5^ At the phylum level, the abundance of Firmicutes was significantly decreased by chronic ethanol feeding while the abundance of Proteobacteria was increased, as compared with that of mice receiving control diet, which was consistent with changes at the family level including the decrease of Ruminococcaceae (*p* < .05) and Lachnospiraceae and the increase of Helicobacteraceae (*p* < .01) and Desulfovibrionaceae (*p* < .01). In the WIP-treated group, the ratio of Firmictues to Proteobacteria as well as the abundance of Lachnospiraceae was effectively elevated in comparison with the vehicle-treated ethanol group (Figure 2d-e). The linear discriminant analysis (LDA) effect size (LEfSe) also demonstrated the increase of Heliocobacteraceae and Desulfovibrionaceae in the ethanol group and the enrichment of Lachnospiraceae in the WIP group (Figure 2f-g). Among the genera significantly changed by ethanol feeding, oral administration with WIP prevented the ethanol-induced reduction of *Blautla, Ruminoclostridum, unidentified_clostridials* and *Alistipes* (Figure 2h-k). In addition, a marked increase of *Bacteroides uniformis* was also observed in the WIP-treated mice. The changes in the gut microbiome of the ethanol-fed mice and the WIP-treated ethanol-fed mice were accompanied with variations in the permeability of intestinal wall and the metabolic endotoxemia. WIP treatment significantly counteracted the alcohol-induced decrease in the tight junction proteins in the ileum (as indicated by the mRNA expression levels of ZO-1 and Occludin) and reduced in the plasma lipopolysaccharide (LPS) (Figure 2l-n). All above results confirm the potential of polysaccharides from *W. cocos* as prebiotic for the treatment of AHS, and support the application of gut microbiota-targeted intervention for ALD.

(a) OTU Venn diagram. (b) Weighted uniFrac-based principal coordinates analysis. (c) Shannon index. Bacterial taxonomic profiling of intestinal bacteria from different groups at the phylum (d) and family level (e). (f, g) Linear discriminant analysis (LDA) scores derived from LEfSe analysis. (h-k) Differentially abundant bacterial genera. The relative expression of occludin-1 (l) and ZO-1 (m) in colon. (n) The level of plasma lipopolysaccharide (LPS). (o) Total fungi in feces assessed by qPCR. The relative expression of ppar-γ (p) and nos2 (q) and colon TNF-α (r) and IL-Iβ (s). (a-k and n) N = 8 per group, (l-s) N = 5 per group. Control: mice received an isocaloric liquid diet instead of ethanol. Alcohol: mice fed with ethanol diet. WIP: mice fed with an ethanol diet supplemented with a water-insoluble polysaccharide from *W. cocos*. Data are presented as the mean ± standard error of the mean (SEM). Statistical analysis was done using one-way ANOVA followed by the Tukey post hoc test. Compare to Alcohol: * *P* < .05; ** *P* < .01.

Due to less knowledge about the contribution of mycobiota to the pathogenesis of AHS, we subsequently focused on the gut fungi in the mice of AHS. In consistent with the early reports,^10,11^ a significantly increased fungal population in the feces of the vehicle-treated ethanol mice was indicated by a quantitative PCR (qPCR) measurement (Figure 2o). Treatment with WIP effectively inhibited the ethanol-induced fungal overgrowth. Hypoxia in the gut restraints the proliferation of *Candida albicans*.^16,17^ The obligate anaerobic bacteria were demonstrated to play a dominant role in maintaining a hypoxic microenvironment by activation of the PPAR-γ signaling via gut butyrate and inhibition of colonic inflammation.^18,19^ Inoculation of *Bacteria thetaiotamicron* and *B. product* in the germ-free mice can prevent the colonization of fungi.^20^ Based on the above results, we deduce that the inactivation of intestinal PPAR-γ signaling due to the alcohol-induced reduction of Firmicutes and Bacteroidetes may be one of the mechanisms underlying the intestinal fungal expansion in animals or patients with ALD. In this study, we observed a downregulated PPAR-γ signaling in the ethanol-fed mice, as indicated by the decrease of PPAR-γ expression and the increase of NOS2 expression (Figure 2p,q), which was correlated with the reductions of bacteria in the Firmicutes and Bacteroidetes. In addition, the pro-inflammatory response in the colon tissues due to the chronic ethanol consumption, as indicated by the increased level of colonic TNF-α and IL-1β (Figure 2r,s), was also supposed to increase the oxygen bioavailability in colonic epithelial cells.^18^ Treatment with WIP dramatically enhanced the abundance of Firmicutes, activated the PPAR-γ signaling, and reduced the inflammation in the colonic epithelia cell, thus preventing the overgrowth of gut fungi and Proteobacteria. The mechanisms for the regulation of fungal growth from the host and other microorganisms in the gut are complicated and need deep investigation.

### *Identification of* Meyerozyma guilliermondii *as a casual fungus for AHS*

**Figure 3.**
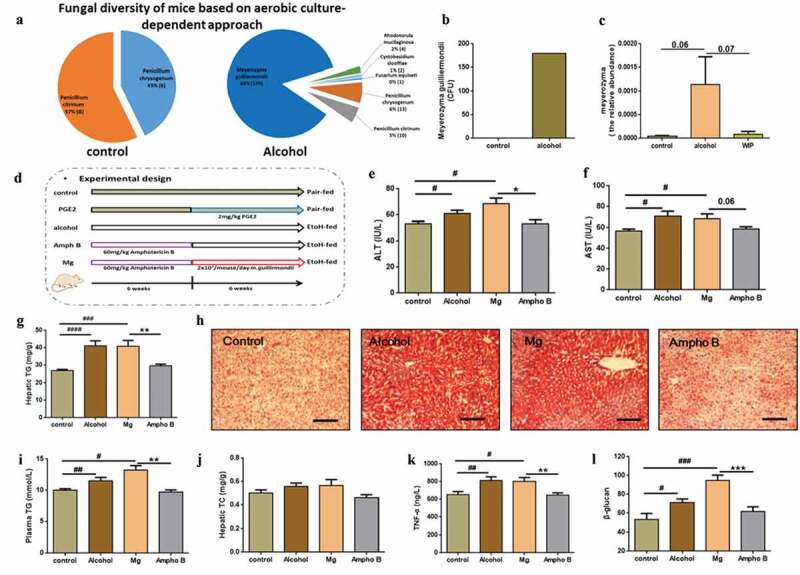
Identification of *Meyerozyma guilliermondii* as a casual fungus for AHS

Next, to find gut fungi directly associating with the development of AHS, we cultured fungal strains in the feces of the ethanol-fed mice by using Dixon agar, the best medium reported in a culturomics method.^21^ A total of 209 strains characterized as *Meyerozyma guilliermondii* (179 strains), *Penicillium chrysogenum* (13 strains), *Penicillium citrinum* (10 strains), *Rhodotorula mucilaginosa* (four strains), *Cystobasidium slooffiae* (two strains), and *Fusarium equiseti* (one strains). In contrast, only 14 strains belonging to *Penicillium chrysogenum* (six strains) and *Penicillium citrinum* (eight strains) were obtained in the fecal sample of mice fed with control diet (Figure 3a-b). The above culturomic data further confirmed the expansion of gut commensal fungi in the mice receiving ethanol, especially the overgrowth of *Meyerozyma guilliermondii*. In addition, an internal transcribed spacer (ITS) sequencing of cecum contents from each group also indicated the enrichment of the gut fungi of *Meyerozyma* in the gut of ethanol-fed mice (Figure 3c, S1). To test whether the overgrowth of *M. guilliermondii* is casually correlated with the development of AHS, we inoculated the amphotericin B-pretreated ethanol mice with the live *M. guilliermondii*. Antifungal treatment with amphotericin B for six weeks completely protected mice from the ethanol-induced damage on liver in the following six weeks, as indicated by the levels of hepatic TG, hepatic TC, plasma TG and liver TNF-α, the activity of ALT and AST, and the degree of lipid deposition shown by oil red O staining (figure 3d-k). However, an aggravated fat accumulation and inflammatory injury were produced when the fungi-free ethanol mice were followingly administrated with *M. guilliermondii* (figure 3d-k). Meanwhile, supplementation with *M. guilliermondii* also increased the level of plasma β-glucan (figure 3l). The above evidence confirmed the contribution of *M. guilliermondii* residing in the gut of mice to the development of AHS.

(a) Fungi isolated from feces by aerobic culture-dependent approach. The number in the parentheses represented the strains obtained for each identified fungus. (b) Level of *Meyerozyma guilliermondii* (Mg) in fecal samples based on aerobic culture-dependent approach. (c) Abundance of *Meyerozyma* in Cecal contents based on ITS1 sequencing. (d) Experimental design. (e) The level of plasma alanine aminotransferase (ALT). (f) The level of plasma aspartate aminotransferase (AST). (g) The level of hepatic triglyceride (TG). (h) Representative picture of liver sections stained with oil-red. (i) The level of plasma triglyceride (TG). (j) The level of hepatic total cholesterol (TC). (k) The level of TNF-α in the liver. (l) The level of plasma β-glucan. (c) N = 4–5 per group, (e-g and i-l) N = 7–9 per group, (h) N = 3 per group. Ampho B: amphotericin B. Data are presented as the mean ± standard error of the mean (SEM). Statistical analysis was done using one-way ANOVA followed by the Tukey post hoc test. Compare to control: # *P* < .05; ## *P* < .01; ### *P* < .001; #### *P* < .0001, Compare to Mg: * *P* < .05; ** *P* < .01, *** *P* < .0001.

### Contribution of fungi-induced PGE_2_ to alcoholic hepatic Steatosis

**Figure 4.**
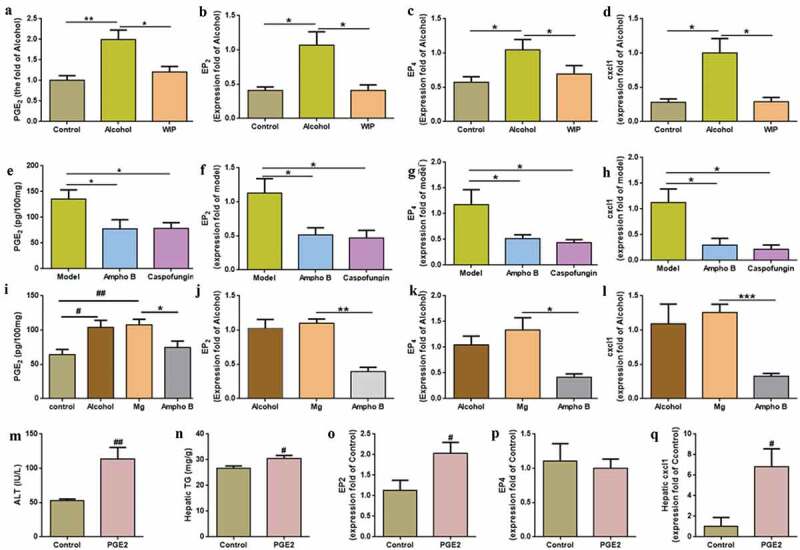
Contribution of fungi-induced PGE_2_ to alcoholic hepatic steatosis

It was reported in rats with an acute hepatic steatosis that the endotoxin-stimulated PGE_2_ production in the liver promoted the triglycerides accumulation via mechanisms dependent on hepatocyte prostaglandin E_2_ (PGE_2_) receptors (EP_2_ and EP_4_).^22^ The expression of the ELR^+^ CXC chemokine CXCL1 (a PGE_2_ downstream target) was also correlated with the neutrophils infiltration in the patients with alcoholic hepatitis.^23^ In this study, chronic ethanol consumption significantly increased the level of hepatic PGE_2_ by 92%, concomitant with the enhanced expression of EP_2_ and EP_4_ and CXCL1 (Figure 4a-d) in the liver. In the WIP-treated AHS mice, the level of PGE_2_ and the expression of EP_2_, EP_4_, and CXCL1 in the liver were greatly reduced (Figure 4a-d), as compared with that of mice of alcohol group.

The level of PGE_2_ (a), the expression of EP_2_ (b), EP_4_ (c) and cxcl1 (d) in liver of ethanol-fed mice treated with WIP. The level of PGE_2_ (e), the expression of EP_2_ (f), EP_4_ (g) and cxcl1 (h) in liver of ethanol-fed mice treated amphotericin B (ampho B) or caspofungin. The level of PGE_2_ (i), the expression of EP_2_ (j), EP_4_ (k) and cxcl1 (l) in liver of the fungi-free mice treated with live *M. guilliermondii* (Mg). The level of ALT (m), hepatic TG (n), the expression of EP_2_ (o), EP_4_ (p) and cxcl1 (q) in liver after oral PGE_2_. (a and m-n) N = 9–10 per group, (b-d, f-h, j-l and o-q) N = 5 per group, (e and i) N = 7–9 per group. Control: mice received the isocaloric liquid diet instead of ethanol. Alcohol: mice fed with ethanol diet. WIP: mice fed with an ethanol diet supplemented with a water-insoluble polysaccharide from *W. cocos*. Ampho B: amphotericin B. Data are presented as the mean ± standard error of the mean (SEM). Statistical analysis was done using one-way ANOVA followed by the Tukey post hoc test. Compare to control: # *P* < .05; ## *P* < .01; Compare to alcohol, model or Mg: * *P* < .05; ** *P* < .01, *** *P* < .001.

In an early report, the gut fungi-induced production of PGE_2_ was attributed to the allergic airway inflammation associated with the gut fungal overgrowth.^13^ To understand whether the increased PGE_2_ in the liver of mice with chronic AHS is correlated with the expansion of gut fungi, mice given a five-week ethanol feeding were orally administrated with ethanol diet supplemented with the non-absorbable amphotericin B, or caspofungin for five weeks (Figure S2A). It was observed that the level of liver PGE_2_ and the expression of EP_2_, EP_4_, CXCL1 were significantly reduced by antifungal treatments (Figure 4e-h), accompanying with the amelioration on the hepatic steatosis (Figure S2B-D). Here, a qPCR analysis also indicated a significant increase in the expression of COX-2, a non-significant enhancement of the microsomal PGE synthase-1 (mPGES-1), and the unchanged expression of COX-1 and cytosolic prostaglandin E2 synthase (cPGEs) in the liver of mice with chronic ethanol feeding. The expression of COX-2 was significantly reduced by antifungal treatments (Figure S4). On the contrary, inoculation of the fungi-free ethanol mice with *M. guilliermondii* enhanced the level of liver PGE_2_ and the expression of EP_2_, EP_4_, and CXCL1 (Figure 4i-l). In addition, mice orally administrated with of PGE_2_ while fed with control diet also showed similar features of alcoholic hepatic steatosis and the increased expression of EP2 and CXCL1 (Figure 4m-q). To further prove the causation of gut fungi-induced PGE_2_ to AHS, the amphotericin B-pretreated ethanol mice were given with the live *M. guilliermondii* while administrating with indomethacin (a PGE_2_ synthase inhibitor). Indomethacin treatment significantly inhibited the influence of *M. guilliermondii* on liver PGE2, EP_2_, EP_4_, and CXCL1, supporting that the gut fungi-induced PGE_2_ production contributes to the development of AHS in mice (Figure S5). Taken together, these results suggest that the gut fungi-induced PGE_2_ production contributes to the development of AHS in mice.

## Discussion

Gut microbiota composed of commensal bacteria, fungi, archaea, and viruses attracts much attention due to its crucial roles in regulating the intestinal homeostasis and host metabolism. Although existing with a relatively low abundance in the gut of human beings, gut mycobiota is attracting increasing attention due to its important functions in keeping health and close association with different diseases including primary sclerosing cholangitis, colorectal cancer, and allergic inflammation.^13,24–26^ Pyrosequencing and culturomics techniques have revealed significant compositional changes in the mycobiome of these diseases. In the setting of alcoholic liver diseases, overgrowth of Candida and the decrease of fungal diversity were found in the patients with different phases of liver diseases covering non-progressive alcoholic liver diseases, alcoholic hepatitis, and alcoholic cirrhosis.^11,27^ In this study, we observed an overgrowth of fungi in the gut of mice with chronic ethanol feeding and further isolated a strain of *M. guilliermondii* that was enriched in the feces of ethanol-fed mice. By using a fungi-free mice model, we confirmed that the enrichment of *M. guilliermondii* in gut aggravated the fatty accumulation and inflammatory damages in liver of the ethanol diet-fed mice. An early study has revealed the presence of *M. guilliermondii* in the fecal samples of healthy people and patients by ITS sequencing and culturomics methods.^21^ The causality and the physiological functions of *M. guilliermondii* in patients with ALD deserve further validation.

Fungal component and metabolite have been causatively associated with the development of AHS.^10,27^ Translocation of fungal β-D-glucan into circulation induced liver inflammation via the CLEC7A receptor.^10^ The candidalysin secreted from *C. albicans* promotes alcohol-associated liver disease by damaging primary hepatocytes.^27^ In the current study, a gut fungi-induced increase of PGE_2_ production in the liver was revealed as one of the mechanisms responsible for the hepatic fat accumulation and inflammatory injury in mice with chronic ethanol feeding. An increased expression of the key enzymes of PGE_2_ synthesis was observed in human NASH livers as compared to controls.^28^ The roles of PGE_2_ in the development of fatty liver diseases are complicated. Some evidence indicated that prostaglandins could contribute to the development of steatosis by enhancing lipid accumulation in liver and suppressing VLDL synthesis and β-oxidation.^29,30^ In other reports, PGE_2_ was demonstrated to reduce the expression of enzymes involved in *de novo* lipogenesis in the liver.^31^ As to the liver inflammatory injury, PGE_2_ was reported to inhibit the production of tumor necrosis factor α (TNF-α) from macrophages and Kupffer cells via EP_2_ and EP_4_ receptors, and thus attenuating the hepatic inflammation.^32,33^ In this study, the increase of liver TNF-α in the mice receiving ethanol could be ascribed to the ethanol-induced LPS overproduction that overwhelmed the anti-inflammatory effect of PGE_2_. In addition, the endogenous PGE_2_ was found to induce MCP-1 expression via EP4 signaling.^34,35^

It was reported that the gut-derived LPS activated the Kupffer cells to produce PGE_2_ in the liver of rats receiving a single large dose of alcohol.^22^ In this work, the significant reduction of PGE_2_ in the liver by antifungal treatment with amphotericin B in combination with the fact that amphotericin B did not change the level of plasma LPS in AHS mice (Figure S2E) conclude that the increased PGE_2_ production in the liver in AHS mice is mainly dependant on gut fungal overgrowth. An early study also confirmed less impact of amphotericin B on ethanol-induced bacterial dysbiosis the intestinal permeability, and the gut barrier function in the ethanol diet-fed mice.^10^

On the other hand, the production of PGE_2_ by fungi is closely associated with fungal virulence and intestinal colonization.^36,37^ PGE_2_ was demonstrated to facilitate the growth of fungi by suppressing the interleukin-17 dependent anti-fungal immunity in mice.^38^ In this work, the gut fungus *M. guilliermondii* enriched in mice with chronic ethanol feeding was found to produce PGE_2_ on culturing with the exogenous arachidonic acid (Figure S3), which might facilitate its intestinal colonization, and contribute to the increase of PGE_2_ in the liver of AHS mice. It is difficult for us to distinguish fungi-produced PGE_2_ and the fungi-activated hepatic PGE_2_ production in this study, but we found that gut fungi-induced PGE_2_ production contributed to about 70% of the PGE_2_ level in the liver of AHS mice based on results obtained in the experiment with amphotericin B. Although the mannan- and β-glucan from *C. albicans* was demonstrated to induce the production of PGE_2_ in the blood mononuclear cell,^39^ the mechanisms involved in the gut fungi-induced hepatic PGE_2_ production remain unknown.

Due to significant pathogenesis contributions from the alcohol-induced microbial dysbiosis and LPS pathway, gut microbiota-related therapies that recover the gut barrier function and integrity, reduce the level of LPS, and inhibit the expansion of commensal fungi are expecting for the treatment of ALD. Indeed, we indicate the water-insoluble polysaccharides from the sclerotium of *W. cocos* is a potential ideal prebiotic meeting these requirements for the treatment of alcoholic hepatic steatosis. Due to the good pharmacological activities and high safety, tests of WIP in clinics deserve further investigation. In summary, we demonstrated that the water-insoluble polysaccharides (WIP) from the sclerotium of W. cocos significantly reduced the inflammatory injury and fat accumulation in the liver of mice receiving chronic ethanol consumption and effectively corrected the gut dysbiosis associated with AHS, supporting WIP as a potential ideal prebiotic for the treatment of AHS. The commensal fungus *M. guilliermondii* that was significantly increased by ethanol feeding while suppressed by WIP was demonstrated to be causatively correlated with AHS. Further mechanism investigation revealed that the ethanol-induced fungal overgrowth in the gut contributed to the development of AHS via the fungi-derived PGE_2_.

## Materials and methods

### Preparation of WIP from Wolfiporia cocos

The polysaccharide WIP was prepared from the fruiting bodies of *W. cocos* and analyzed by methods as described in our early study. The purity of WIP was verified by high performance gel permeation chromatography (HPGPC) analysis. The structural characteristics of WIP were determined to be a (1-3)-β-D-glucan with an average Mw of 4.486 × 10^6^ Da by NMR and SEC-RI-MALLS analyses.^14^

### Animal care and experiments

All procedures were performed in accordance with recommendations in the Guide for the Care and Use of Laboratory Animals of the Institute of Microbiology, Chinese Academy of Sciences (IMCAS) Ethics Committee (Permit No. APIMCAS2017023). C57BL/6 J mice were purchased from the Experimental Animal Center, Chinese Academy of Medical Sciences. In our early research, an oral administration of WIP at the dose of 1.0 g/kg was demonstrated to alleviate hepatic steatosis in *ob/ob* mice through enhancing the abundance of butyrate-producing gut bacteria. Thus, we investigated the effect of WIP on alcoholic hepatic steatosis at the dose of 1.0 g/kg (the dose equivalent to 0.11 g/kg in human) in this study.

To study the influence of WIP supplementation in alcoholic hepatic steatosis, 8-week-old C57BL/6 J male mice were fed with a gradual increase in ethanol concentrations from 0% to 4% (v/v) from day 1 to day 5 during the acclimatization stage, and then fed with 4% (v/v) ethanol-containing Lieber-DeCarli liquid diet for five weeks. After determining the liver injury by alanine transaminase (ALT) and Aspartate aminotransferase (AST) activity at the fifth week, mice were divided into two groups. One group (WIP group) is orally administrated with WIP at a dose of 1.0 g/kg/day for another five weeks while continuing with 4% ethanol (v/v)-containing Lieber-DeCarli liquid diet. The other group (alcohol group) is given with an equal volume of water while fed with 4% ethanol (v/v) containing Lieber-DeCarli liquid diet. Ten mice receiving isocaloric liquid diet instead of ethanol was used as control.^40^

To the experiment for antifungal treatment on mice with AHS, the method is the same as WIP. Non-absorbable antifungal drugs amphotericin B (60 mg/kg/d) or caspofungin (5 mg/kg/d) was orally administrated for five weeks.

To prove the effect of *M. guilliermondii* (Mg) and PGE_2_ on alcoholic hepatic steatosis, C57BL/6 male mice were sorted into five groups: control (control diet + 0.2 mL sterilized water), PGE_2_ (control diet + oral administration of PGE_2_ at the dose of 2 mg/kg/day), alcohol (ethanol diet + 0.2 mL sterilized water), Mg (ethanol diet + oral administration of amphotericin B at the dose of 60 mg/kg/d for the first six weeks, and then *M. guilliermondii* at the dose of 2*10^7^/day for the following six weeks), Ampho B (ethanol diet + oral administration of amphotericin B at the dose of 60 mg/kg/day for the first six weeks, and then 0.2 mL sterilized water for the following six weeks).

To mechanistically link fungi and PGE_2_, we designed an inhibition experiment in vivo. C57BL/6 male mice were sorted into three groups: alcohol (ethanol diet + 0.2 mL sterilized water), Mg (ethanol diet + oral administration of amphotericin B at the dose of 60 mg/kg/d for the first two weeks, and then *M. guilliermondii* at the dose of 2*10^7^/day for the following six weeks), Mg+In (ethanol diet + oral administration of amphotericin B at the dose of 60 mg/kg/d for the first two weeks, and then *M. guilliermondii* at the dose of 2*10^7^/day and indomethacin at the dose of 4 mg/kg/d for the following six weeks)

### Tissue sampling

After treatment, animals were anesthetized with diethyl ether and blood was sampled from the portal and cava veins. After exsanguination, mice were euthanized by cervical dislocation. Subcutaneous adipose tissue deposits, intestines, and liver were precisely dissected, weighed, immediately immersed in liquid nitrogen, and stored at −80°C for further analysis.

### Biochemical analyses

The levels of plasma alanine transaminase (ALT), aspartate aminotransferase (AST), triglyceride (TG), and total cholesterol (TC) were measured by a commercial kit (Nanjing Jianchen Bioengineering Institute, Jiangsu, China). The liver homogenate was prepared from the fresh liver tissue in the physiological saline (10%, w/v). Then, the homogenate was centrifuged at 3000 rpm for 10 min at 4°C. The supernatant was kept for further analysis. Hepatic triglycerides (TG) and total cholesterol (TC) in the supernatant were quantified with above commercially available kit.

### Real-time qPCR analysis

Total RNA was extracted from the liver tissue with the TRIzol reagent according to the manufacturer’s protocol (Invitrogen, Carlsbad, CA, USA). Reverse transcription was performed on 1 μg of total RNA using a cloned AMV first-strand cDNA synthesis kit (Tianjin, Beijing, China). Primers used for cDNA amplification by real-time PCR are listed in Table S1. Glyceraldehyde-3-phosphate dehydrogenase (GAPDH) was used as the housekeeping gene for normalization of the target genes expression. PCR reactions were performed using Perfecta SYBR green super mix (KAPA).

### Microbial community analysis

**(i) Bacteria**. Total DNA was isolated from cecum content, sequencing the variable V3 and V4 regions of the 16S rRNA gene was performed with the IonS5^TM^XL platform. The 16S rRNA gene V3-V4 region was amplified using the primers 341 F (CCTAYGGGRBGCASCAG) and 806 R (GGACTACNNGGGTATCTAAT). After shear filtration of reads, an average of 74,339 reads was measured per sample. A total of 69,871 effective data were obtained through quality control with the effective rate of 94.03%. The reads were assigned to operational taxonomic units (OTUs) with a 97% similarity threshold, and a total of 673 OTUs were identified. The species annotation of OTUs sequences was conducted with silva132 databases. Data were analyzed as described in our early report.^41^

**(ii) Fungi**. Cecum contents were suspended in 50 mM Tris buffer (pH7.5) containing 1 mM EDTA, 0.2% β-mercaptoethanol (Sigma) and 1000 U/ml of lyticase (Sigma). The mix was incubated at 37°C for 30 min and fungal genomic DNA was extracted by using the CTAB method. The fungal microbiome was analyzed by sequencing of internal transcribed spacer (ITS). Here, we used a two-step nested PCR method for amplification. Firstly, the ITS fragments were amplified by the primers ITS1 (TCCGTAGGTGAACCTGCGG) and ITS4 (TCCTCCGCTTATTGATATGC) using a KAPA HiFi HotStart Ready Mix (Kapa Biosciences, Wilmington, MA). Briefly, for each sample, 2 μL genomic DNA was used for a 25 μl PCR under the following conditions: 95°C for 5 min, 35 cycles of 45 s at 95°C, 55°Cfor 45s, and 72 for 1 min, followed by 7 min at 72°C. Then, the amplicon was diluted for 2nd PCR using the primers 1737 F (GGAAGTAAAAGTCGTAACAAGG) and 2043 R (GCTGCGTTCTTCATCGATGC). The purified amplicon were constructed for single-end sequencing based on the IonS5^TM^XL sequencing platform. The reads were assigned to operational taxonomic units (OTUs) with a 97% similarity threshold and taxonomy assignment of the resulting OTU was carried out using the BLAST against the UNITE reference database. The sequencing data of bacteriome and mycobiome in this work had been public on gcMeta had been public on gcMeta [ref: https://doi.org/10.1093/nar/gky1008] with projectID NMDC10013102 (https://gcmeta.wdcm.org/opendata/opendataDetail/d4c1012282e111ea92a7b49691092464/) which can be accessed through ftp://bio-mirror.im.ac.cn/public_data/d20200420_liuhongwei.

### Culture of fungi

Gut fungi in the fecal sample were cultured using an early-described method.^21^ 100 mg of mixed fecal sample from three mice in the control or alcohol group was diluted with 900 μL of phosphate buffer saline (PBS) for solid and liquid fungal culture. 1/10th dilutions were performed. A 50 μL of each dilution was used for culturing on Dixon agar (DIX) solid culture media supplemented with 3 antibiotics; namely, colistin (30 mg/L), vancomycin (30 mg/L) and imipenem (30 mg/L).

### Molecular fungal identification

Direct ITS analysis was performed for fungal species isolates. The ITS1 (TCCGTAGGTGAACCTGCGG) and TIS4 (TCCTCCGCTTATTGATATGC) primers were used for PCR and sequencing. The fungus was identiﬁed on the basis of morphology and the DNA sequences of the ITS regions of their ribosomal RNA gene. Analysis of sequences showed homology with that of *Meyerozyma guilliermondii* (99.64%, accession number: KP675394.1), *Penicillium chrysogenum* (100%, accession number: MH151126.1), *Penicillium citrinum* (100%, accession number: KU216720.1), *Rhodotorula mucilaginosa* (99.65%, accession number: KP960512.1), *Cystobasidium slooffiae* (99.64%, accession number: MK386939.1), and *Fusarium equiseti* (99.21%, accession number: KY426410.1) in GenBank.

### Measurement of PGE_2_ production

*M. guilliermondii* was grown in Potato Dextrose Broth (PDB) for 48 h, and then cultures were added with 500 mM arachidonic acid (AA) and shaken at 28°C for 24 h. Supernatants were then analysis by PGE_2_ ELISA kit (Cayman Chemical). To assay the level of PGE_2_ in the liver, the frozen liver was pulverized in a stainless steel cylinder cooled with liquid nitrogen. The powdered liver was immediately added to 2.0 mL of 80% ethanol and then mixed well. After centrifugation at 5000 rpm for 10 min at 4°C, the supernatant was extracted using SPE columns (Phenomenex, Torrance, CA). Columns were pre-washed with 2.0 mL of MeOH followed by 2.0 mL of H_2_O. After applying the sample, the columns were first eluted with 1.0 mL of 10% MeOH followed by 1 mL of MeOH. The eluent of MeOH was dried under nitrogen and then re-dissolved in assay buffer. PGE_2_ levels were determined with PGE_2_ ELISA kit according to manufacturer’s instructions.

### Statistical analysis

All results are expressed as mean ± SEM. For multiple comparisons, statistical analysis was performed using one-way or two-way ANOVA followed by the Tukey’s multiple comparison tests with GraphPad 6.0.

## Supplementary Material

Supplemental Material
