# Supplementary material for: Therapeutic manipulation of gut microbiota by polysaccharides of Wolfiporia cocos reveals the contribution of the gut fungi-induced PGE2 to alcoholic hepatic steatosis
Source: Gut Microbes. 2020 Oct 27;12(1):1830693. doi: 10.1080/19490976.2020.1830693 (PMC7592601; doi:10.1080/19490976.2020.1830693)
Supplement: Supplemental Material [file KGMI_A_1830693_SM5322.docx]

**Therapeutic manipulation of gut microbiota by polysaccharides of *Wolfiporia cocos* reveals the contribution of the gut fungi-induced PGE_2_ to alcoholic hepatic steatosis**

Shanshan Sun ^a, b, #^, Kai Wang^a, #^, Li Sun^a, c^, Baosong Cheng^a, c^, Shanshan Qiao^a, c^, Huanqin Dai^a^, Wenyu Shi^d^, Juncai Ma^d^, Hongwei Liu ^a, b, c*^

^a^ State Key Laboratory of Mycology, Institute of Microbiology, Chinese Academy of Sciences, Beijing 100101, China

^b^ School of Life Sciences, University of Science and Technology of China, No. 96 Jinzhai-Road, Hefei 230026, P. R. China

^c^ Savaid Medical School, University of Chinese Academy of Sciences, Beijing, 100049, P. R. China.

^d^ Microbial Resource and Big Data Center, Institute of Microbiology, Chinese Academy of Sciences, Beijing 100101, China

^#^ SS. Sun and K. Wang contributed equally to this article.

* Corresponding Author Tel: +86 10 64806074; Email: liuhw@im.ac.cn.

**Table S1. qPCR primer sequences for the targeted mouse genes.**

| Primers | Forward Sequence | Reverse Sequence |
| --- | --- | --- |
| TNF-α | TAGCCAGGAGGGAGAACAGA | TTTTCTGGAGGGAGATGTGG |
| ppar-γ | GCAGCTACTGCATGTGATCAAGA | GTCAGCGGGTGGGACTTTC |
| nos2 | AACAATTCCTGGCGTTACCTT | TGTATTCCGTCTCCTTGGTTC |
| ptger2 | GAGGTTTCATCCATGTAGGCA | AGAGGAGAGAGGACTTCGATG |
| ptger4 | CTGATGTCTTTCACCACGTTTG | CATCTTACTCATCGCCACCTC |
| cxcl1 | TGCACCCAAACCGAAGTCAT | TTGTCAGAAGCCAGCGTTCAC |
| gapdh | TGTAGACCATGTAGTTGAGGTCA | AGGTCGGTGTGAACGGATTTG |
| COX-2 | TGGGTGTGAAGGGAAATAAGG | CATCATATTTGAGCCTTGGGG |
| mPGES-1 | AGGATGCGCTGAAACGTGGAG | CCGAGGAAGAGGAAAGGATAG |
| COX-1 | TCCCTGAGATCTGGACCTGGC | TGAGTACTTCTCGGATGAAGG |
| cPGES | AAGGAGGTGACCGAGTTTTGC | GAGAGATGAGATGCACCAGCC |


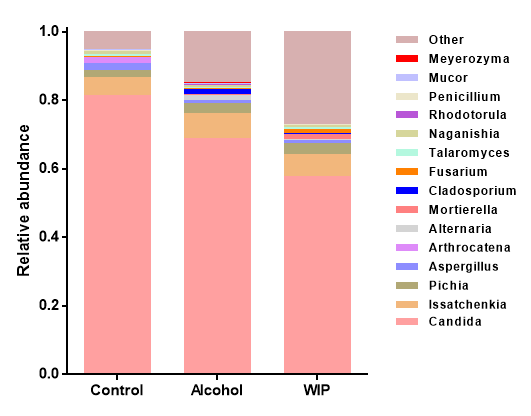


**Figure S1. ITS1 sequencing of cecum samples from C57BL/6 mice.**


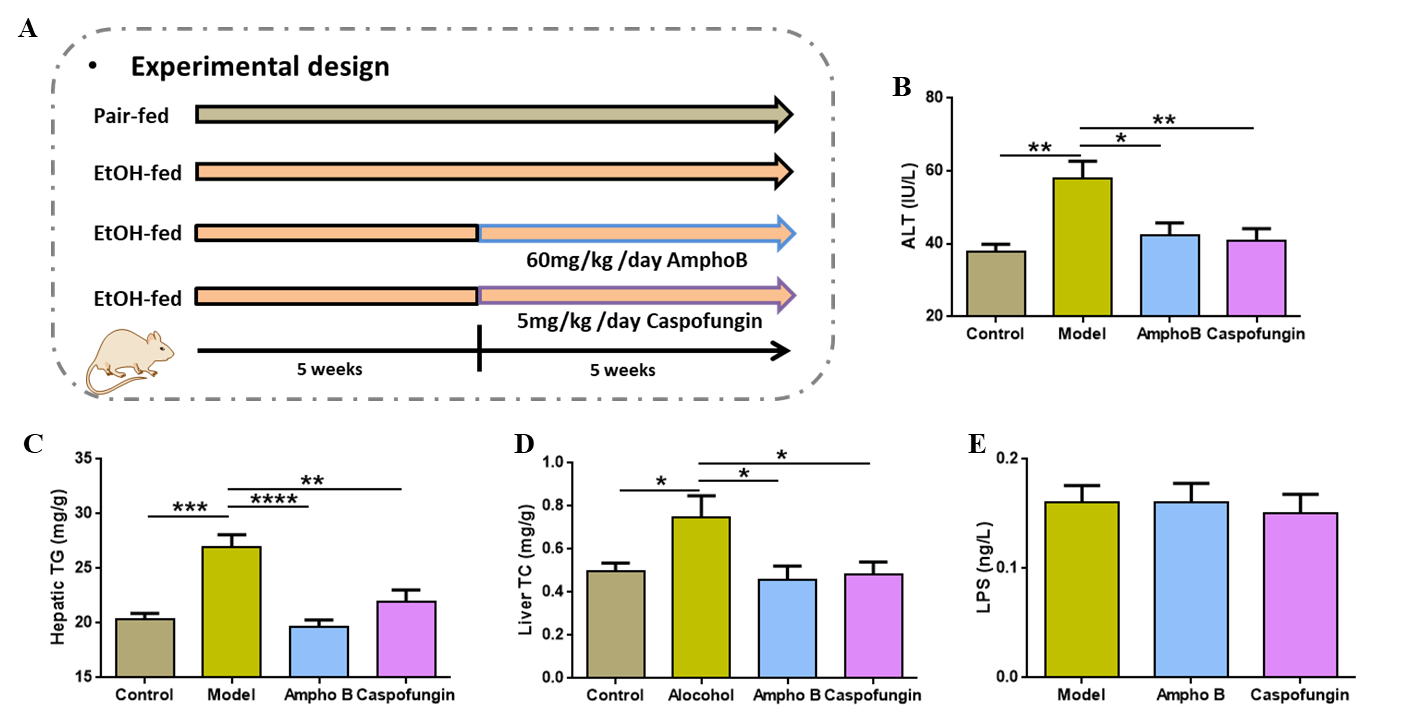


**Figure S2. Decreased ethanol-induced liver disease in mice treated with antifungals.**

(A) experimental design. (B) Plasma levels of aspartate aminotransferase (AST). (C) Hepatic triglyceride (TG). (D) hepatic total cholesterol (TC). (E) Plasma Lipopolysaccharide (LPS). (B-E) N=8 per group. Data are presented as the mean ± standard error of the mean (SEM). Statistical analysis was done using one-way ANOVA followed by the Tukey post hoc test. Compare to model: * P<0.05; ** P<0.01, *** P<0.001, **** P<0.0001.


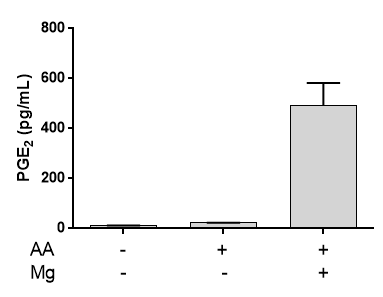


**Figure S3. Production of PGE2 by in vitro culture of *M. guilliermondii* (Mg)**

*M. guilliermondii* isolated from mouse feces was cultured in the presence of 500 mM arachidonic

acid and assayed for PGE2 in the culture supernatant.


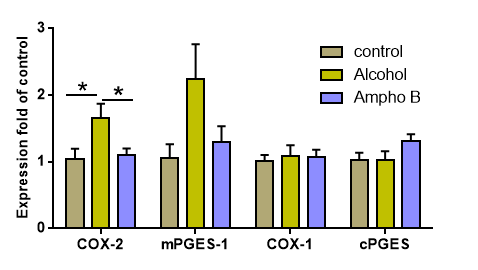


**Figure S4. Expression of COX and PGES in liver as determined by qPCR (N = 5 per group).**


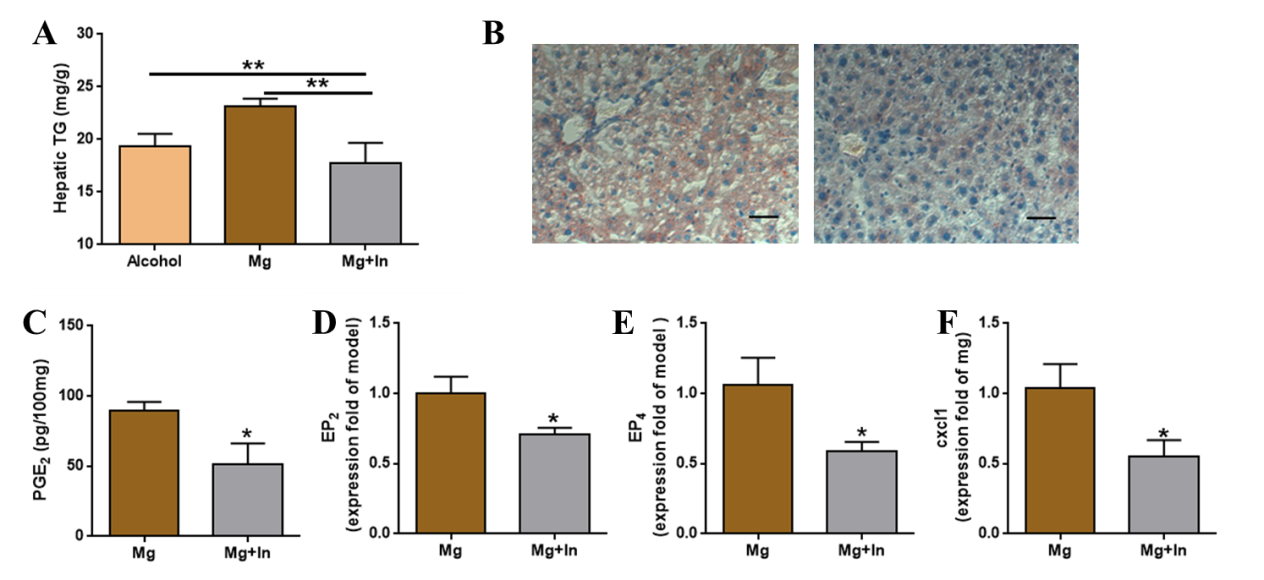


**Figure S5. Indomethacin inhibited the influence of *M. guilliermondii* on liver**

(A) Hepatic triglyceride (TG). (B) Liver sections of oil-red staining. The level of PGE_2_ (C), the expression of EP_2_ (D), EP_4_ (E) and cxcl1 (F) in liver. (A, C) N=7-9 per group, (B) N=3 per group, (D-F) N=5 per group. Data are presented as the mean ± standard error of the mean (SEM). Statistical analysis was done using one-way ANOVA followed by the Tukey post hoc test.* P<0.05; ** P<0.01

*Meyerozyma guilliermondii* with similarity of 99.64% to that of KP675394.1 in the GeneBank

TACGAATTCTTTTGCGCGCTTACTGCGCGGCGAAAAACCTTACACACAGTGTCTTTTTGATACAGAACTCTTGCTTTGGTTTGGCCTAGAGATAGGTTGGGCCAGAGGTTTAACAAAACACAATTTAATTATTTTTACAGTTAGTCAAATTTTGAATTAATCTTCAAAACTTTCAACAACGGATCTCTTGGTTCTCGCATCGATGAAGAACGCAGCGAAATGCGATAAGTAATATGAATTGCAGATTTTCGTGAATCATCGAATCTTTGAACGCACATTGCGCCCTCTGGTATTCCAGAGGGCATGCCTGTTTGAGCGTCATTTCTCTCTCAAACCCCCGGGTTTGGTATTGAGTGATACTCTTAGTCGGACTAGGCGTTTGCTTGAAAAGTATTGGCATGGGTAGTACTGGATAGTGCTGTCGACCTCTCAATGTATTAGGTTTATCCAACTCGTTGAATGGTGTGGCGGGATATTTCTGGTATTGTTGGCCCGGCCTTACAACAA

CCAAACAAGTTTGACCTCAAATCAGGTAGGAATACCCGCTGAACTTAAGCATATCTAAGAGGGGGGGGAAAAT

*Penicillium chrysogenum* with similarity of 100% to that of MH151126.1 in the GeneBank

TGAGGGCTCTGGGTCCACCTCCCACCCGTGTTTATTTTACCTTGTTGCTTCGGCGGGCCCGCCTTAACTGGCCGCCGGGGGGCTTACGCCCCCGGGCCCGCGCCCGCCGAAGACACCCTCGAACTCTGTCTGAAGATTGTAGTCTGAGTGAAAATATAAATTATTTAAAACTTTCAACAACGGATCTCTTGGTTCCGGCATCGATGAAGAACGCAGCGAAATGCGATACGTAATGTGAATTGCAAATTCAGTGAATCATCGAGTCTTTGAACGCACATTGCGCCCCCTGGTATTCCGGGGGGCATGCCTGTCCGAGCGTCATTTCTGCCCTCAAGCACGGCTTGTGTGTTGGGCCCCGTCCTCCGATCCCGGGGGACGGGCCCGAAAGGCAGCGGCGGCACCGCGTCCGGTCCTCGAGCGTATGGGGCTTTGTCACCCGCTCTGTAGGCCCGGCCGGCGCTTGCCGATCAACCCAAATT

*Penicillium citrinum* with similarity of 100% to that of KU216720.1 in the GeneBank

AGCGGGACCTCGGGGCCAACCTCCCACCCGTGTTGCCCGAACCTATGTTGCCTCGGCGGGCCCCGCGCCCGCCGACGGCCCCCCTGAACGCTGTCTGAAGTTGCAGTCTGAGACCTATAACGAAATTAGTTAAAACTTTCAACAACGGATCTCTTGGTTCCGGCATCGATGAAGAACGCAGCGAAATGCGATAACTAATGTGAATTGCAGAATTCAGTGAATCATCGAGTCTTTGAACGCACATTGCGCCCTCTGGTATTCCGGAGGGCATGCCTGTCCGAGCGTCATTGCTGCCCTCAAGCCCGGCTTGTGTGTTGGGCCCCGTCCCCCCCGCCGGGGGGACGGGCCCGAAAGGCAGCGGCGGCACCGCGTCCGGTCCTCGAGCGTATGGGGCTTCGTCACCCGCTCTAGTAGGCCCGGCCGGCGCCAGCCGACCCCCAACCTTTAATTATCTCAGGTTGACCTCGGATCAGGTAGGGA

*Rhodotorula mucilaginosa* with similarity of 99.65% to that of KP960512.1 in the GeneBank

TGGGAACGTCCACTTAACTTGGAGTCCGAACTCTCACTTTCTAACCCTGTGCACTTGTTTGGGATAGTAACTCTCGCAAGAGAGCGAACTCCTATTCACTTATAAACACAAAGTCTATGAATGTATTAAATTTTATAACAAAATAAAACTTTCAACAACGGATCTCTTGGCTCTCGCATCGATGAAGAACGCAGCGAAATGCGATAAGTAATGTGAATTGCAGAATTCAGTGAATCATCGAATCTTTGAACGCACCTTGCGCTCCATGGTATTCCGTGGAGCATGCCTGTTTGAGTGTCATGAATACTTCAACCCTCCTCTTTCTTAATGATTGAAGAGGTGTTTGGTTTCTGAGCGCTGCTGGCCTTTACGGTCTAGCTCGTTCGTAATGCATTAGCATCCGCAATCGAACTTCGGATTGACTTGGCGTAATAGACTATTCGCTGAGGAATTCTAGTCTTCGGACTAGAGCCGGGTTGGGTTAAAGGAAGCTTCTAATCAGAATGTCTACATTTTAAGATTAGATCTCAAATCAGGTAGGACTACCCGCTGAACTTAAGCATATCAATAAAGCGGAGGAAT

*Cystobasidium slooffiae* with similarity of 99.64% to that of MK386939.1 in the GeneBank

ACGGATTTAGGACGTTCTTTTTAGAAGTCCGACCCTTTCATTTTCTTACACTGTGCACACACTTCTTTTTTACACACACTTTTAACACCTTAGTATAAGAATGTAATAGTCTCTTAATTGAGCATAAATAAAAACAAAACTTTCAGCAACGGATCTCTTGGCTCTCGCATCGATGAAGAACGCAGCGAATTGCGATAAGTAATGTGAATTGCAGAATTCAGTGAATCATCGAATCTTTGAACGCACCTTGCACTCTTTGGTATTCCGAAGAGTATGTCTGTTTGAGTGTCATGAAACTCTCAACCCCCCTATTTTGTAATGAGATGGGTGTGGGCTTGGATTATGGTTGTCTGTCGGCGTAATTGCCGGCTCAACTGAAATACACGAGCAACCCTATTGAAATAAACGGTTTGACTTGGCGTAATAATTATTTCGCTAAGGACGTTTTCTTCAAATATAAGAGGTGCTTCTAATTCGCTTCTAATAGCATTTAAGCTTTAGACCTCAAATCAGTCAGGACTACCCGCTGAACTTAAGCATATCAATAAAGCGGGAGGAAC

*Fusarium equiseti* with similarity of 99.21% to that of KY426410.1 in the GeneBank

AAGAGCTTACCTCCAACCCCTGTGACATACCTATACGTTGCCTCGGCGGATCAGCCCGCGCCCCGTAAAACGGGACGGCCCGCCCGAGGACCCCTAAACTCTGTTTTTAGTGGAACTTCTGAGTAAAACAAACAAATAAATCAAAACTTTCAACAACGGATCTCTTGGTTCTGGCATCGATGAAGAACGCAGCAAAATGCGATAAGTAATGTGAATTGCAGAATTCAGTGAATCATCGAATCTTTGAACGCACATTGCGCCCGCCAGTATTCTGGCGGGCATGCCTGTTCGAGCGTCATTTCAACCCTCAAGCTCAGCTTGGTGTTGGGACTCGCGGTAACCCGCGTTCCCCAAATCGATTGGCGGTCACGTCGAGCTTCCATAGCGTAGTAATCATACACCTCGTTACTGGTAATCGTCGCGGCCACGCCGTTAAACCCCAACTTCTGAATGTTGACCTCGGATCAGGTAGGAATACCCGCTGAACTTAAGCATATCATAAAGAGCGGAGGAAAA
